# cnvCapSeq: detecting copy number variation in long-range targeted resequencing data

**DOI:** 10.1093/nar/gku849

**Published:** 2014-09-16

**Authors:** Evangelos Bellos, Vikrant Kumar, Clarabelle Lin, Jordi Maggi, Zai Yang Phua, Ching-Yu Cheng, Chui Ming Gemmy Cheung, Martin L. Hibberd, Tien Yin Wong, Lachlan J. M. Coin, Sonia Davila

**Affiliations:** 1 Department of Genomics of Common Disease, School of Public Health, Imperial College London, London W12 0NN, UK; 2 Genome Institute of Singapore, 60 Biopolis St., 138672, Singapore; 3 Institute of Medical Molecular Genetics, University of Zurich, Wagistrasse 12, 8952 Schlieren, Switzerland; 4 Singapore Eye Research Institute, Singapore National Eye Center, 11 Third Hospital Avenue, 168751, Singapore; 5 Department of Ophthalmology, National University of Singapore, 1E Kent Ridge Road, 119228, Singapore; 6 Faculty of Infectious and Tropical Diseases, London School of Hygiene and Tropical Medicine, London WC1E 7HT, UK; 7 Institute for Molecular Bioscience, University of Queensland, St Lucia, QLD 4072, Australia

## Abstract

Targeted resequencing technologies have allowed for efficient and cost-effective detection of genomic variants in specific regions of interest. Although capture sequencing has been primarily used for investigating single nucleotide variants and indels, it has the potential to elucidate a broader spectrum of genetic variation, including copy number variants (CNVs). Various methods exist for detecting CNV in whole-genome and exome sequencing datasets. However, no algorithms have been specifically designed for contiguous target sequencing, despite its increasing importance in clinical and research applications. We have developed cnvCapSeq, a novel method for accurate and sensitive CNV discovery and genotyping in long-range targeted resequencing. cnvCapSeq was benchmarked using a simulated contiguous capture sequencing dataset comprising 21 genomic loci of various lengths. cnvCapSeq was shown to outperform the best existing exome CNV method by a wide margin both in terms of sensitivity (92.0 versus 48.3%) and specificity (99.8 versus 70.5%). We also applied cnvCapSeq to a real capture sequencing cohort comprising a contiguous 358 kb region that contains the Complement Factor H gene cluster. In this dataset, cnvCapSeq identified 41 samples with CNV, including two with duplications, with a genotyping accuracy of 99%, as ascertained by quantitative real-time PCR.

## INTRODUCTION

In the post-genomic era, next-generation sequencing (NGS) has revolutionized biological research and discovery. Despite its relatively short history, NGS has been universally adopted as the standard for exploring genomic variation. However, it still remains economically infeasible to use whole-genome sequencing (WGS) in the large sample sizes that are needed to identify rare variants of small effect or incomplete penetrance. Thus, targeted resequencing is being used as a cost-effective alternative to WGS for investigating regions of interest when a priori knowledge of potentially causal loci is available.

Targeted resequencing strategies, including whole-exome sequencing (WES), have been used to elucidate both monogenic (1–3) and complex disorders (4–6), including some cancers (7–9). These studies, however, tend to focus exclusively on single-nucleotide variants (SNVs) and indels, essentially disregarding structural variation. Structural variants, and copy number variants (CNVs) in particular, have been shown to contribute significantly to genetic diversity (10) and disease etiology (11–13). The scarcity of CNV findings obtained from targeted resequencing can be largely attributed to systematic biases that arise from the target selection process and render traditional, whole-genome CNV detection algorithms inapplicable.

Multiple approaches have been developed to selectively enrich for specific genomic loci prior to sequencing. However, in addition to known sequencing biases, the enrichment step, whether PCR- or hybridization-based, unavoidably introduces non-uniformity in sequencing coverage across the target regions. This translates into highly variable read depth (RD) that is not directly proportional to the underlying copy number of the region, as assumed by most CNV detection methods developed for WGS. Even CNV algorithms that rely more heavily on discordant paired reads (14,15) rather than RD are adversely affected by the variable capture specificity which results in insert sizes that are not readily interpretable. Furthermore, such methods are only effective if the CNV breakpoints are successfully captured, which can be challenging in homologous genomic regions. To overcome these difficulties, a few CNV detection methods have been specifically developed for targeted resequencing.

The vast majority of these methods employ two basic strategies to deal with enrichment biases: control-based normalization or data-driven normalization. Control-based normalization attempts to counteract local sequencing artifacts by dividing the RD by a control depth, thus generating a log-ratio metric. Existing methods in this category include ExomeCNV (16), EXCAVATOR (17) and CONTRA (18), which require either a matched control population that is not always available or an unrealistic pseudo-control calculated from the population average. Data-driven normalization, on the other hand, attempts to identify and eliminate high-variance components in the RD signal that are likely dominated by noise. This strategy essentially constitutes a dimensionality reduction that can be achieved either using Singular Value Decomposition (SVD), or the equivalent Principal Component Analysis (PCA). Popular methods in this category include CoNIFER (19) and xHMM (20). Post-normalization, all the aforementioned methods perform CNV detection using either simple thresholding or established CNV segmentation algorithms such as Circular Binary Segmentation (CBS) and Hidden Markov Models (HMM). Furthermore, regardless of normalization strategy, the ability to call absolute copy number genotypes currently requires the use of a control population.

All of the existing methods focus on detecting exon-spanning CNVs in WES datasets and are based on the assumption that RD correlation across distal regions reflects sample batch artifacts. As a result, they require exome-wide data and cannot accurately resolve CNV breakpoints outside exons, which makes them unsuitable for long-range contiguous capture sequencing. A subset of the existing algorithms, comprising CONTRA, CoNIFER and xHMM, can theoretically accommodate large capture regions, but not without significant modifications beyond the scope of their intended use.

CONTRA corrects for correlated noise between samples using a control or pseudo-control population. Since it was originally designed for small-region targeted resequencing, CONTRA relies on heuristics for predicting large CNVs. CoNIFER and xHMM perform a z-transformation of raw RD signal, followed by SVD and PCA respectively. Both methods exclude samples and probes of high-variance and attempt to avoid over-correction using empirical rules. All three methods rely exclusively on RD and none of them provide absolute genotypes. Another significant obstacle presented by all three methods is the fact that they require a list of capture target coordinates as input. This is readily available for commercial WES platforms, but not for custom contiguous long-range capture assays.

Here we present cnvCapSeq, a control-free method for accurate and sensitive CNV discovery and absolute copy-number genotyping in long-range targeted resequencing datasets. cnvCapSeq is the first algorithm specifically designed to address the challenges of contiguous capture sequencing. By utilizing information at the population level, our method ameliorates the effects of capture efficiency bias and minimizes the risk of over-correction without the need for a baseline reference. Unlike existing methods, cnvCapSeq integrates evidence from both RD and read pairs (RP) to achieve high breakpoint resolution regardless of coverage uniformity.

## MATERIALS AND METHODS

### Samples and datasets

cnvCapSeq was developed and benchmarked using an extensive multi-locus simulation dataset, designed to cover a wide range of scenarios. Our framework was then validated on a real cohort of 285 Chinese Singaporean control samples. The real cohort comprises contiguous capture sequence data of the Regulation of Complement Activation (RCA) locus on chromosome 1.

### Simulations

To obtain a comprehensive benchmark for cnvCapSeq, we generated a synthetic dataset that spans multiple CNV lengths and population frequencies. For that purpose, we selected 21 genomic loci on chromosomes 1 and 6 (Supplementary Table S1) that were shown to harbor recurrent deletions by the 1000 Genomes Project (21). The sizes of the candidate deletions are evenly distributed between 1 and 115 kb. To facilitate CNV calling, each locus includes 20 kb on either side of the candidate deletion.

We used Wessim (22) to perform in silico simulations of contiguous capture sequencing reads in the 21 candidate loci. Although Wessim was originally designed for exome sequencing simulations, it can be generalized for any type of capture sequencing experiment. By emulating a probe-hybridization step, Wessim generates very realistic synthetic capture data that cannot be obtained with traditional NGS simulators. To take advantage of this feature, we fragmented the candidate loci into unevenly spaced probes with random overlaps, and queried the reference genome (hg19) for the corresponding probe sequences. These sequences constitute the input for Wessim. However, the probe design itself is obscured from later analysis, as it would be in real capture experiments. The random overlap of the hybridization probes approximates the custom probe tiling that is necessary for comprehensive coverage of a target region and is largely responsible for the observed uneven coverage. The second source of RD bias within capture targets is the presence of repetitive genomic sequence that exhibits low alignability. Such non-unique sequence has been shown to mediate CNV formation, and may therefore be over-represented in CNV regions. This type of artifact can only be reproduced by considering candidate loci with known CNVs, instead of simulating under ideal conditions. The complex structure of our 21 loci, poses alignability challenges and renders our synthetic dataset more realistic (Supplementary Figure S1).

In each locus we simulated 100 copy-neutral samples to aid in specificity and precision calculations and 30 samples with heterozygous deletions to evaluate sensitivity. An empirical error model was used to simulate paired-end reads from Illumina HiSeq IIx sequencing runs. The reads were 100 bp long with a mean insert size of 200 bp, and were aligned to the reference using the Burrow-Wheeler Aligner (BWA) (23). The average coverage was kept constant at 200x across samples with 94.5% of all bases (across loci) covered at least 10x. Bases with coverage below 10x were excluded from further analysis, as they correspond to targets that failed to capture. This is most likely due to low alignability of the underlying probes.

Finally, the deletion-carrying samples were combined with the copy-neutral samples to obtain pseudo-populations of varying deletion frequencies (1–30%) in each locus. These pseudo-populations form the basis for evaluating the performance of cnvCapSeq against CONTRA, CoNIFER and xHMM.

### RCA cohort

The RCA cohort comprises capture sequence data of a ∼358 kb locus on chromosome 1q31.3 that contains the Complement Factor H (*CFH*) gene and 5 CFH-related (CFHR) genes. Although this region is known to harbor a common deletion and has previously been associated with Age-related Macular Degeneration (AMD) (24) and susceptibility to meningococcal disease (25), it remains difficult to characterize due to its high degree of macrohomology.

The target region was enriched using a custom Nimblegen SeqCap assay. Despite the highly repetitive nature of the RCA locus, the assay successfully captured ∼90% of the intended 358 kb region (chr1:196 620 597–196 978 814). In each assay 24 samples were captured, all of which were uniquely barcoded. Due to the relatively small size of the target region, two 24-sample capture libraries could be multiplexed to allow sequencing of 48 samples per flow cell on Illumina HiSeq2000, using a paired-end protocol. The sequencing libraries consisted of 100 bp reads with a mean insert size of 200 bp. The sequencing reads were aligned to the human reference genome (hg19) using CASAVA, which is part of Illumina's data analysis pipeline. Duplicate reads were removed using Picard followed by local realignment and recalibration with GATK (26,27). The average depth of coverage across samples is ∼650x and more than 90% of bases were covered at least 14x (Supplementary Figure S2). This is expected since the assay did not contain baits to capture the remaining 10%.

### Read depth normalization

**Figure 1.**
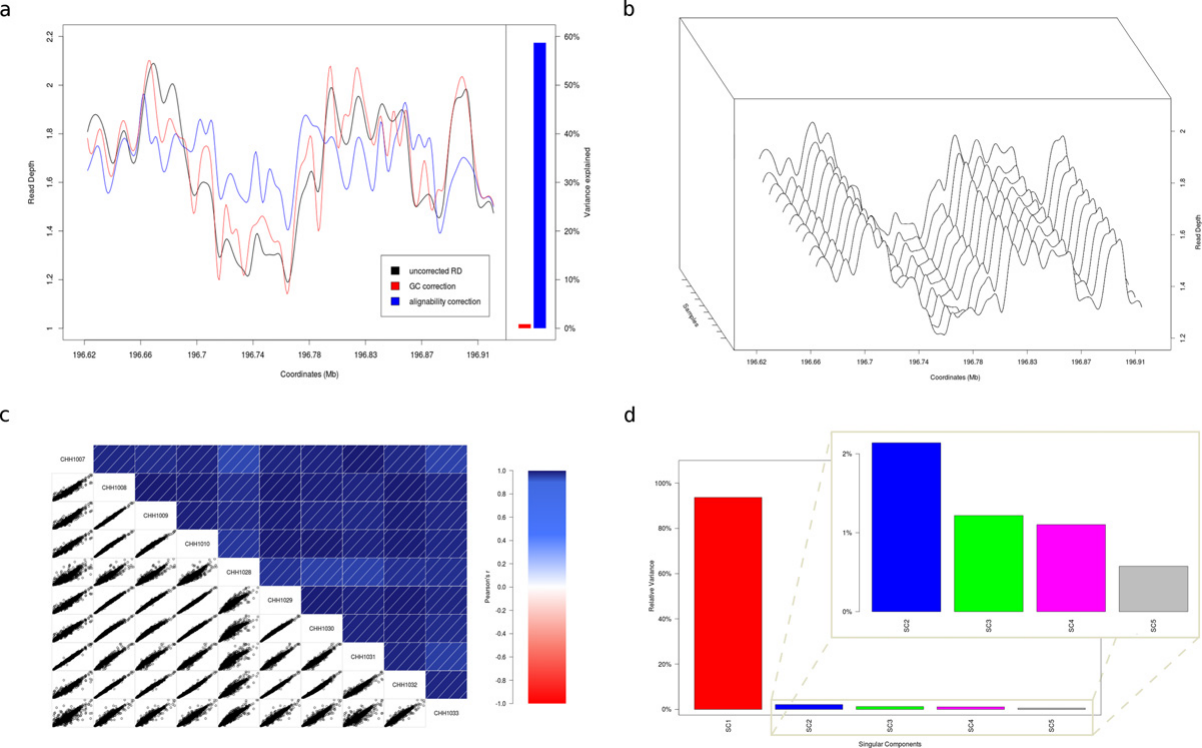
Read depth profiles. (**a**) Read depth profile of sample CHH1030 (RCA cohort) across the target region, before and after correcting for GC-content and alignability using linear regression. The coefficient of determination (*R*^2^, ratio of explained variation to the total variation) is used to determine the variance explained by each variable. GC explains only a small fraction of the read depth variance, while alignability explains 57%. (**b**) Read depth profile for 10 randomly chosen samples from the RCA cohort across the target region. The profiles appear to have a high degree of similarity, with only minor variations between samples. (**c**) Correlogram for the 10 samples in (b). The lower left panel represents the pair-wise scatterplot for all possible sample combinations. The upper right panel represents the color- and intensity-coded Pearson's correlation (*r*^2^) for all possible sample combinations. The minimum reported *r*^2^ is 0.93. (**d**) The relative variance for the first five singular components calculated from our entire dataset. The relative variance of component *k* is defined as *s*_*k*_^2^/*Σ_i_s*_*i*_^2^. The first singular component dominates with a relative variance of 93%, while the remaining components have minor contributions.

The main source of systematic bias arises from varying capture efficiency, which leads to uneven coverage across the target region. Capture efficiency depends on various factors, including sequence composition and enrichment strategy. Especially for hybrid capture technologies, the bait length and tiling density play an essential role in the resulting RD pattern. Most aspects of sequence composition, such as GC content and alignability, are well described and can thus be modeled (28). Sequence properties, however, account for only a small fraction of the noise present in capture datasets (Figure 1a).

Bait design, on the other hand, may explain most of the variability in RD (Supplementary Figure S1) but is usually proprietary and as such remains a black box for normalization purposes. Although the individual confounders of sequencing coverage may remain unknown, it is apparent that the noise pattern is consistent among samples (Figure 1b; Supplementary Figure S1; Supplementary Figure S3). In fact, the RD is highly correlated across samples, with an average pair-wise Pearson's correlation coefficient of 0.97 (Figure 1c). Therefore, RD measurements are highly amenable to data-driven normalization, which is especially powerful for large-scale population resequencing projects.

The SVD has been proposed as a robust mathematical framework for detecting high-order structure in complex biological datasets (29). Assuming our RD data is sampled in non-overlapping windows of user-defined length (default 100 bp), normalized by the average per-sample coverage and arranged into a position-by-sample matrix *M*, SVD provides the following factorization:

\begin{equation*} {M} = {U}\Sigma {V}^* \end{equation*}

The columns of matrix *U* represent the left singular vectors of *M* which can be interpreted as uncorrelated eigen-windows. Similarly, the rows of V* represent the right singular vectors of *M* and can be thought of as uncorrelated eigen-samples. *Σ* is a diagonal matrix containing the singular values of *M*, in decreasing order. The magnitude of each singular value corresponds to the relative importance of each combination of eigen-window and eigen-sample and is largely dependent on the number of samples being processed in parallel.

**Figure 2.**
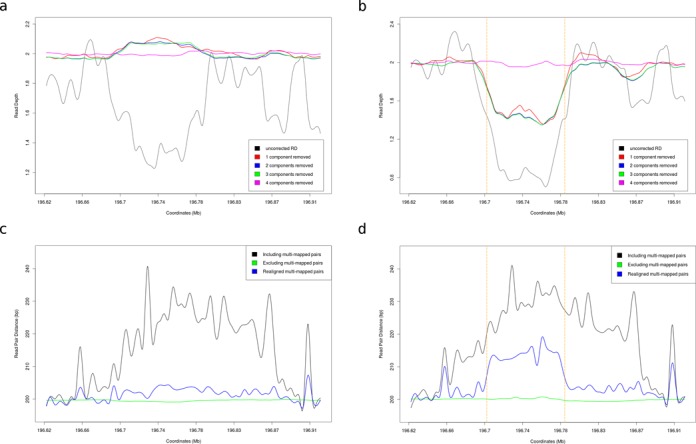
Normalization results for a copy-neutral sample and a sample with CNV from the RCA cohort. (**a**) Read depth profile for sample CHH1008 with up to four singular components removed. This sample is copy-neutral (as validated by qPCR). (**b**) Read depth profile for sample CHH1039 with up to four singular components removed. This sample contains a PCR-validated heterozygous deletion (with breakpoints marked by the orange dashed lines). (**c**) RP Distance profile for sample CHH1008 with various strategies for dealing with multi-mapped reads. (**d**) RP Distance profile for sample CHH1039. By locally realigning multi-mapped reads we manage to unmask a relatively weak RP signal that supports the deletion.

Existing methods that apply SVD or PCA for RD normalization in exome sequencing datasets (19,20) use heuristics to determine the number of components that need to be removed. In our case, however, it was evident that the first singular value is dominated by the systematic noise (Figure 1d; Supplementary Figure SA). Thus, by removing only the first singular component and reconstructing the M matrix, we essentially remove the baseline coverage, making CNV signal stand out (Figure 2a and b; Supplementary Figure S3b and e).

Also, unlike other SVD-based methods, we don't convert our reconstructed data into *z*-scores, since that eliminates all scale information and allows only relative copy number estimation. Instead, we add the mean coverage back to the normalized data, thus restoring its original properties. The shifted, normalized RD can then be treated like its unnormalized counterpart, providing an intuitive basis for absolute copy number genotyping.

### Read pair processing

RP provide distinct CNV signatures that are largely complementary to RD. When the distance of a mapped RP is significantly different from the expected insert size, it can be used to identify both deletions and duplications. Unlike RD, however, RP signatures only arise when the sequencing targets happen to contain CNV breakpoints. Since exome probes are unlikely to capture such breakpoints, RPs have been largely overlooked by targeted resequencing CNV methods.

The analysis of RP data becomes even more challenging in the presence of repeats and segmental duplications, such as those found in the RCA locus. As a result, we observe a large proportion of multi-mapped pairs with ambiguous distances that are essentially uninformative for CNV calling. By entirely excluding such reads, as CNV algorithms usually do, all RP information appears to be lost. Instead, we attempt to rescue non-unique mappings using sensitive local realignment. This allows us to unveil weak RP signatures that support the absence (Figure 2c) or presence of CNV (Figure 2d).

To that end, we extract discordant multi-mapped RP and perform very sensitive local realignment using Bowtie 2 (30). To increase alignment sensitivity we adjust Bowtie's multiseed heuristic by reducing the length of seeds and the inter-seed distance, while increasing the number of permitted mismatches per seed. Thus, we obtain multiple alternative mappings with detailed alignment properties, which may have been sacrificed by the original aligner in favor of speed. We then rank the alternative mappings according to their reported AS and YS alignment scores and select the combination that maximizes their sum while minimizing the overall amount of soft-clipping. Pairs with alternative mappings of indistinguishable quality are filtered out from further analysis. The rest are used to calculate the average distance of all spanning pairs at any given position (RPS; read pair span), along with a count of such pairs (RPC; read pair count). To account for possible differences in library design among samples, we quantile-normalized the insert size distributions to a Gaussian reference with a mean of 200 and 15 bp variance. Finally, these metrics are sampled at the same resolution as the RD.

### Data modeling and CNV calling

Considering the contiguous nature of our sequencing target, the assay likely captures CNV breakpoints thus allowing us to model both RD and RP at the population level to achieve optimal results. CNV detection and genotyping is achieved using the HMM framework described in cnvHiTSeq (31). The observed continuous variables (RD, RPS and RPC) are considered to be generated by the hidden underlying discrete copy number states.

For WGS, the HMM emission probabilities of RD and RPC were modeled using the negative binomial (NB) distribution, whereas a normal distribution was used for the RPS. However, given that our normalization strategies reduce the dynamic range of both RD and RP, we tailored the distributions for capture sequencing datasets. Thus, we adopted a fine-tuned set of initial emission parameters for both the NB and the normal distribution, combined with a higher initial transition rate, to increase the sensitivity of the model without affecting its specificity.

Another important addition to the CNV calling framework is the ability to a priori exclude regions that correspond to failed target probes or intentional gaps. If there is information on loci that were not captured during the enrichment step, cnvCapSeq will downweight all evidence arising from these loci, thus avoiding spurious CNV calls.

### cnvCapSeq implementation

cnvCapSeq is implemented as a collection of Java tools and helper shell scripts for UNIX systems. It takes BAM alignment files as input and offers the option to exclude target regions that are known to have failed the capture step (in BED format). The pre-processing of the BAM files is performed with SAMtools (32), while Bowtie2 is used to realign multi-mapped reads. cnvCapSeq generates normalized RD files in binary and text format that can be used independently of our HMM framework. Thus, cnvCapSeq also offers a standalone normalization tool that can be used in conjunction with third party segmentation algorithms. When paired with our HMM solution, cnvCapSeq produces CNV calls in text format and optional segmentation plots.

The sampling density of RD and RPC is a user-specified parameter that determines the CNV breakpoint resolution and the computational requirements of the algorithm. At the default high-resolution setting of 100 bp, the analysis of a ∼350 kb region in 100 samples (sequenced at 50x), requires 4 GB of memory and one CPU hour. The software is freely available at http://sourceforge.net/p/cnvcapseq.

### Experimental validation

cnvCapSeq's results on the RCA cohort were validated using quantitative real-time PCR (qPCR) on a randomly chosen subset of the samples with predicted CNVs, in duplicates and whenever possible in triplicates. The subset comprised 13 samples, representing at least one sample from each detected CNV pattern. qPCR was also performed on 10 additional samples in which CNV was not identified. We designed four sets of primers distributed across the entire 358 kb region (Supplementary Table S2). Using all sets of primers for each CNV gave a reasonable estimate on its length. For instance, the most frequently detected CNV overlaps through two primer sets, located in the beginning and the end of the CNV, but not the remaining two sets. To confirm that the primers were targeting the intended region, we performed PCR followed by Sanger sequencing for one sample. For internal control a fifth set of primers for *PRKG1* (a house keeping gene) was also created. Following qPCR, copy number estimates were obtained using the ΔΔCt method of relative quantification.

## RESULTS

### Simulation benchmark

A systematic evaluation of cnvCapSeq's performance was obtained using our extensive simulation dataset. This dataset can serve as a general benchmark for contiguous capture sequencing algorithms and is thus made publicly available. For comparison purposes, we also included three representative WES CNV methods in our assessment: CONTRA, CoNIFER and xHMM. However, the applicability of these methods to contiguous targets is limited by their explicit requirement for capture target coordinates. The first obstacle for obtaining such coordinates is the proprietary nature of bait design for custom capture assays. Furthermore, the assumption of contiguity is contrary to the distinct nature of exons and genes spread across the genome. We attempted to overcome these constraints by generating ‘pseudo-targets’ that cover our simulated loci. This was achieved by dividing the loci into non-overlapping 100 bp windows.

Our simulation dataset explores numerous conditions and normalization scenarios. Before proceeding with the detailed assessment, however, we investigated how the normalization process itself affects our synthetic RD data. To that end, we applied the SVD and observed that most of the variance that is due to unmeasurable sources of experimental bias, can be captured by the first and largest singular value (Supplementary Figure S3). In most cases, the absence of CNV in copy-neutral samples only becomes apparent after removing the first singular component (Supplementary Figure S3a and d). Conversely, true CNVs are masked by an overlapping reduction in RD that is present in all samples (Supplementary Figure S3b and e). Removing more than one singular component masks all evidence of CNV (Supplementary Figure S3c and f) and renders RD uninformative. Thus, we opted to normalize our simulated data by eliminating just the first singular component. The other data-driven methods in our benchmark (xHMM and CoNIFER) employ heuristics to determine the number of components to remove, which results invariably in over-correction. Therefore, we decided to override the default behavior of these methods, forcing them to discard only the first singular component. This creates bias in favor of xHMM and CoNIFER, but allows for meaningful comparison with cnvCapSeq.

First, we calculated overall performance metrics to obtain a broad view across simulated conditions. In this assessment, cnvCapSeq outperforms all other methods by a wide margin (Supplementary Table S3), with an overall sensitivity of 92.0 versus 48.3% for the next best method (xHMM) and an overall specificity of 99.8 versus 70.5%. cnvCapSeq also has a clear advantage in positive prediction value (PPV), as it achieves 98.4 versus 12.3% for xHMM. This disparity is due to the fact that all three WES CNV methods make considerably more false positive calls than true positive calls. In fact, CoNIFER detected almost none of the simulated deletions (0.02% sensitivity) and it was thus excluded from further comparisons.

**Figure 3.**
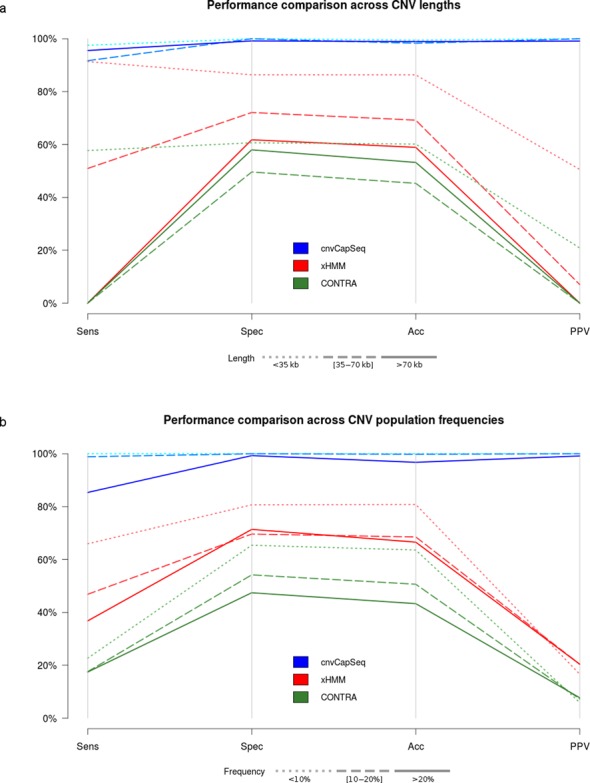
Performance comparison for three CNV detection methods using the synthetic dataset. Parallel coordinates plot represent combinations of sensitivity, specificity, accuracy and positive predictive value (PPV). (**a**) Simulated deletions were divided into three size groups and the performance metrics were averaged across frequencies. The denser the dash pattern the larger the underlying deletions. The best results for all methods are achieved for shorter CNVs (**b**) Simulated deletions were divided into three frequency groups and the performance metrics were averaged across deletion sizes. The denser the dash pattern the higher the underlying frequencies. The best results for all methods are achieved for lower frequencies.

Next, we set out to explore the relationship between the length of the simulated CNV and method performance. Our synthetic dataset comprises 21 genomic loci, each harboring a deletion of different size. The deletions range from 1014 to 114 663 bp, with an approximate increment of 5 kb. cnvCapSeq remains consistently specific and precise across lengths while exhibiting small variations in sensitivity. xHMM and CONTRA, however, appear to deteriorate significantly with increasing CNV size. The effect is more pronounced for CNVs larger than 70 kb, which both xHMM and CONTRA fail entirely to detect (Figure 3a, Supplementary Figure S4). This can be largely explained by the fact that WES methods were designed for small- to medium-sized CNVs, spanning neighboring exons. Thus, they resort to heuristics for calling larger events, which tend to restrict their functionality.

In capture sequencing datasets, CNV detection is contingent on the ability to generate a robust RD baseline that represents the copy-neutral state across targets. Whether explicitly required (as in control-based normalization) or indirectly estimated (as in data-driven approaches), this baseline is largely affected by the population frequency of the underlying CNV. We tested this effect by simulating a wide range of CNV population frequencies (1–30%), while maintaining a constant overall population size of 100. As expected, higher frequencies pose challenges for all methods, with xHMM more severely affected (Figure 3b). cnvCapSeq maintains 100% sensitivity for frequencies up to 15%, while specificity and PPV only start to decline at frequencies higher than 27%. On the other hand, xHMM and CONTRA start deteriorating for frequencies as low as 6% (Supplementary Figure S5).

Finally, we investigated how the size of the cohort influences CNV detection. Small cohorts may be economically preferable, but tend to suffer from higher variance, which leads to unreliable estimates of the RD baseline. This is especially problematic for dimensionality-reducing techniques, which attempt to compensate by eliminating more singular (or principal) components and discarding high-variance probes. This comes at the expense of true CNV signal, which often lies in regions with unstable RD. In contrast, cnvCapSeq employs a conservative filtering approach, which confers enhanced performance for smaller datasets and increased robustness to sample size. This was demonstrated by simulating various cohort sizes (5–100 samples), while keeping the CNV population frequency approximately equal to 30%. In this challenging scenario, cnvCapSeq is shown to outperform the next best method (xHMM) by a wider margin for small datasets than for the full-size cohort (Supplementary Figure S6). Moreover, cnvCapSeq reaches stability for cohorts comprising as few as 40 samples, while xHMM requires twice as many samples for optimal results (Supplementary Figure S6).

Thus, we have demonstrated that cnvCapSeq is superior to methods designed for exome CNV detection under a variety of conditions. cnvCapSeq also overcomes a major limitation of existing data-driven normalization methods, which can only detect rare variants, while requiring concurrent analysis of large sample numbers.

### RCA cohort

cnvCapSeq was also evaluated in a real cohort comprising targeted resequencing data from the ∼350 kb RCA locus in 285 control samples. The RCA locus lies on chromosome 1q and contains the CFH gene along with five ancestrally related genes that arose through duplication of CFH. The samples were normalized in three batches, corresponding to the flow cells they were sequenced on, and then pooled for CNV analysis.

We normalized the RD using our SVD framework and investigated how the results differ from our simulated dataset. We verified our previous observation regarding the contribution of singular components to the RD noise profile (Figure 1d). As in our simulated data, we observe a highly correlated RD pattern across samples, which swamps the true CNV signal. Both the absence (Figure 2a) and the presence of CNV (Figure 2b) are elucidated when the first singular component is removed and suppressed when removing higher-order components. Therefore, by filtering out only the first singular component we eliminate the noise pattern, exposing relatively weaker RD perturbations caused by CNV.

We also subsampled the RCA cohort to assess the consistency of our normalization approach for smaller datasets. Thus, we confirmed that our strategy can help expose common CNVs (up to 30% population frequency) in pseudo-cohorts comprising as few as five samples (Supplementary Figure S7).

Discordant RP provide orthogonal evidence for the presence of CNV and have been shown to greatly improve the specificity of CNV detection algorithms when combined with RD (31). Nevertheless, RP remain underutilized in targeted resequencing experiments, since they are only relevant when the size of the target is larger than the insert size and the CNV breakpoints are captured. RP distance profiles are also confounded by multi-mapped reads. Rescuing such ambiguous pairs is especially important in our case, since the common deletion in the RCA gene cluster is facilitated by nonallelic homologous recombination between the same repeat elements that also give rise to the multi-mappings (33). Therefore, excluding such alignments would eliminate all RP evidence for the deletion we are aiming to detect (Figure 2c and d).

**Figure 4.**
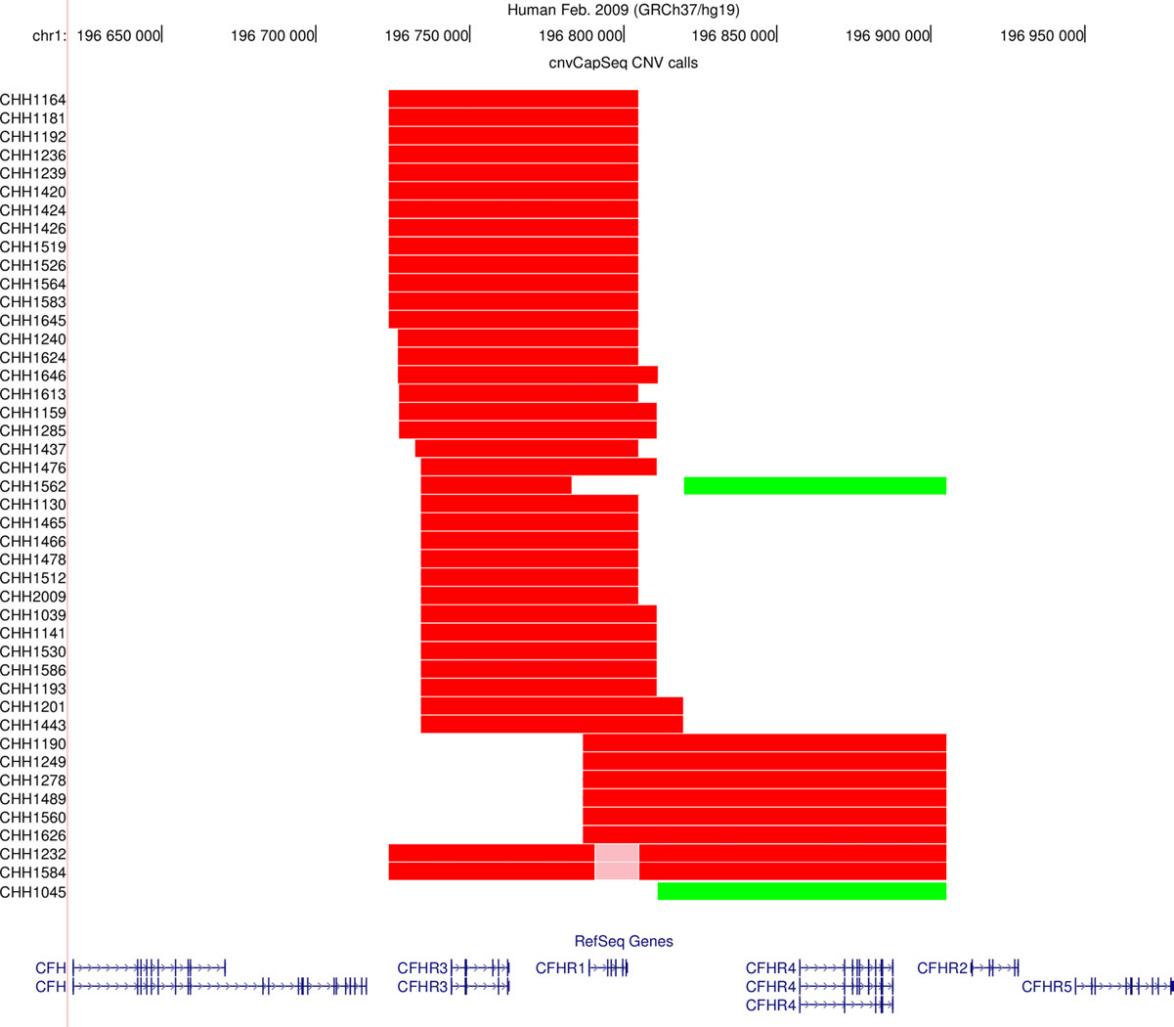
Graphical representation of our CNV calls. The UCSC genome browser was used to plot the CNVs detected by cnvCapSeq along with the affected genes. Red color denotes heterozygous deletion, pink denotes homozygous deletion and green corresponds to three copies.

Following normalization, we performed CNV segmentation and genotyping using a HMM. In our control dataset, cnvCapSeq detects 42 CNVs in 41 samples (Figure 4), most of which are consistent with the common ∼80 kb deletion that has been previously reported in the RCA locus (24,33) and results in the loss of CFHR1 and CFHR3. We also report an ∼90-kb duplication that affects CFHR4 in two samples. Finally, we identified a 120-kb heterozygous deletion that overlaps both the common deletion and the duplication, in eight samples (Supplementary Table S4, Supplementary Figure S8).

### RCA benchmark

First, we set out to validate our CNV calls using qPCR. To that end, we genotyped 13 of the predicted CNVs, as well as 10 copy-neutral samples. cnvCapSeq achieved a perfect concordance (13 out of 13 CNV calls) with the PCR results, indicating very high precision (Table 1a). Furthermore, no false positives were detected in the copy-neutral samples, corresponding to high specificity (Table 1b). In addition, all the CNV lengths were predicted to be accurate at the locus level, except for a duplication which was predicted to be shorter by cnvCapSeq than the qPCR estimate. This corresponds to an average genotyping accuracy of 99% across the four primer sets.

**Table 1.**
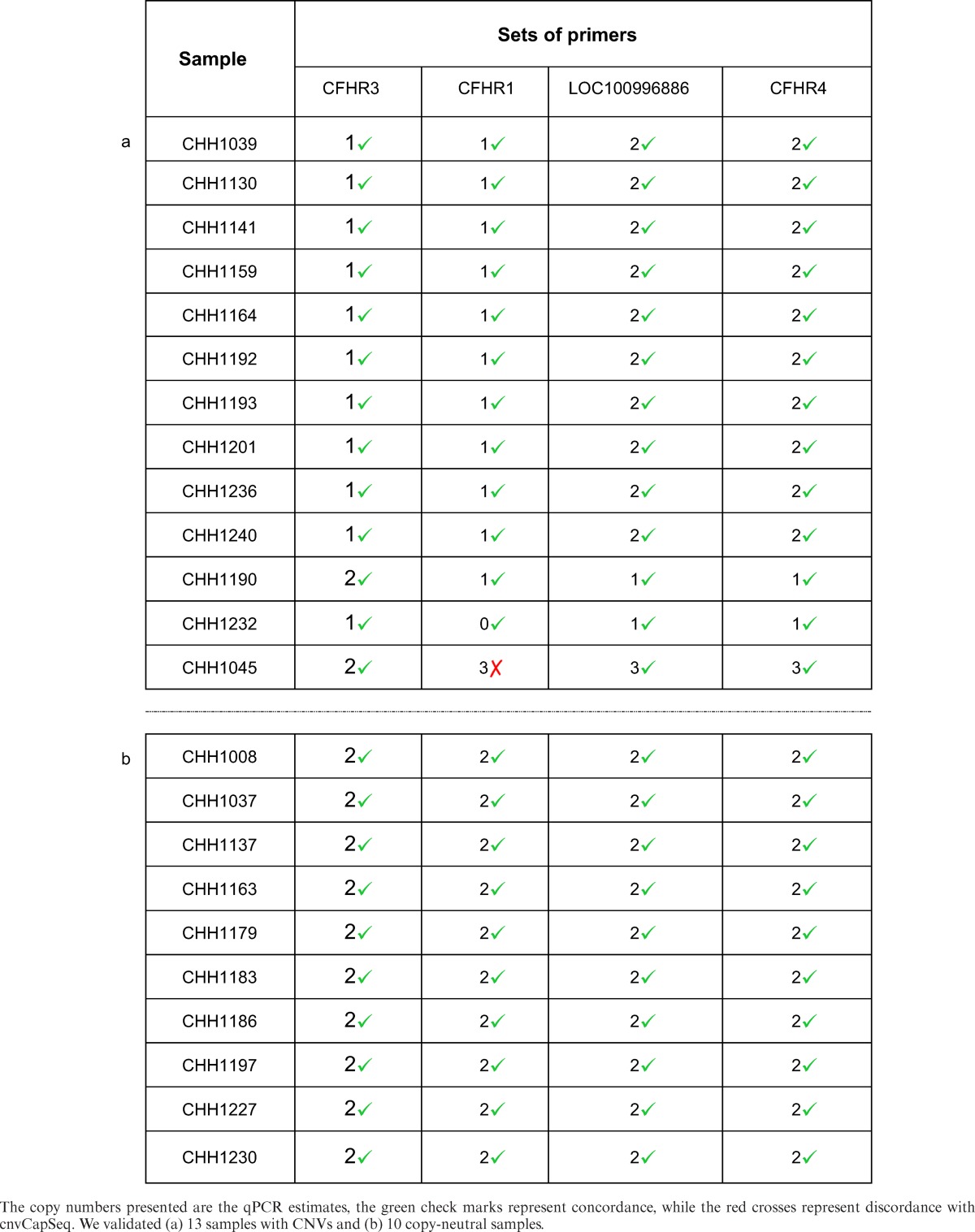
qPCR validation results

Next, we compare cnvCapSeq's performance against CONTRA, CoNIFER and xHMM. These methods were designed for exome sequencing datasets and therefore require capture target coordinates as input. Thus, we followed a similar process to our simulation benchmark by dividing our 358 kb capture region into non-overlapping 100 bp windows which serve as ‘pseudo-targets’.

We run CONTRA on our pseudo-target dataset using the entire population to create the required pseudo-control. CONTRA has a special set of parameters for predicting large CNVs using CBS. Even using these parameters, however, CONTRA identified 28 short CNVs (300 bp–5 kb) that overlap neither the PCR results nor any of the remaining cnvCapSeq calls. By increasing the significance threshold we were able to recover three of the PCR-validated deletions at the expense of four validated false positives (and 134 CNVs in samples that were deemed copy-neutral by cnvCapSeq).

xHMM applies a strict filtering approach to exclude samples and target probes that exhibit high variance. It also employs an empirical rule to select how many principal components to eliminate. Using the default parameters, most samples failed quality control and the subsequent removal of eight components produced no CNV calls. When we omitted the variance filtering step and forced the removal of a single principal component, xHMM detected 24 CNVs. These CNVs overlap 19% of cnvCapSeq callset and include only two out 13 qPCR validated results and one validated false positive.

CoNIFER, has a similar recommendation for high-variance samples, which we ignored as it would have eliminated more than half of our dataset. CoNIFER's fundamental difference from xHMM is that it requires visual inspection of the scree plot to determine the number of components to be removed. Based on this empirical approach we identified eight components for elimination, which again failed to produce any CNVs. When we relaxed this criterion to the minimum recommended value of three components, CoNIFER identified 58 variants, consisting mostly of duplications. Out of the 13 PCR-validated CNVs, CoNIFER successfully detected only three and had one false positive. When we forced CoNIFER to remove only the first singular component, the overall number of calls doubled, the number of validated CNVs rose to four, but the number of false positives also increased to three.

Finally, we wanted to investigate how the sequencing depth of coverage affects our method's performance. Our original RCA dataset had highly variable coverage, ranging from 300x to 1400x across samples. To establish a minimum threshold for coverage, we randomly downsampled our data to 50x and 10x and rerun the analysis. The results for 50x were almost indistinguishable from the original, except for minor breakpoint differences. Using a 50% reciprocal overlap criterion, 100% of the downsampled CNV calls agreed with the unconstrained analysis. Even at 10x, which is on the lowest end for targeted resequencing experiments, cnvCapSeq correctly identified most CNVs, albeit with slightly underestimated lengths (Supplementary Figure S9). The only exceptions were the misclassification of a PCR-validated nested, homozygous deletion as heterozygous and the fragmentation of a large deletion into two smaller ones. This led to 93% of the 10x CNV results overlapping the original callset.

## DISCUSSION

Targeted resequencing technologies offer a cost- and time-efficient alternative to WGS and are thus rapidly gaining in popularity. Exome sequencing is the most common form of targeted resequencing, as it focuses on the genome-wide analysis of protein-coding variants. However, when the biological hypothesis instructs a more focused approach, larger contiguous regions are targeted in an effort to identify intronic and regulatory variants. CNVs in particular, can be detected more reliably and in higher resolution with larger targets, but there's been a paucity of compatible algorithms. To that end, we have presented cnvCapSeq, a dedicated framework for discovery and genotyping CNVs in large-target capture resequencing datasets.

cnvCapSeq has distinct advantages compared to existing CNV detection methods for capture sequencing. Since it was designed for large contiguous regions, our algorithm doesn't require a priori knowledge of capture target coordinates. Furthermore, it is the only capture-specific method to incorporate evidence from discordant RP, which become relevant as the target size increases. cnvCapSeq doesn't standardize or transform the underlying data and can thus generate absolute copy numbers without a reference panel. We have demonstrated that cnvCapSeq achieves a high accuracy along with high precision, without the need for matched control samples. Our method is also robust to high allele frequencies, low depths of coverage and to high coverage variability within datasets.

As with all dimension-reducing techniques, cnvCapSeq gains power by concurrently analyzing multiple samples. Unlike current methods, however, cnvCapSeq avoids eliminating true signal by removing only a single singular component from RD. The potential trade-off is higher residual noise, which is counterbalanced using RP for increased specificity and HMM for spatial smoothing. Thus, cnvCapSeq can be used to analyze smaller datasets and genotype both rare and more common variants.

cnvCapSeq was tested on a Nimblegen SeqCap assay, but remains agnostic to the enrichment technique, requiring only BAM alignment files as input. Thus, our method's data-driven normalization approach is applicable in principle to all hybridization-based targeted sequencing of contiguous regions, regardless of platform. However, enrichment protocols that don't rely on hybrid capture may exhibit distinct properties and biases beyond the scope of cnvCapSeq.

## AVAILABILITY

The software and the simulated dataset are freely available at http://sourceforge.net/p/cnvcapseq.

## SUPPLEMENTARY DATA

Supplementary Data are available at NAR Online.

SUPPLEMENTARY DATA
