# Supplementary material for: cnvCapSeq: detecting copy number variation in long-range targeted resequencing data
Source: Nucleic Acids Res. 2014 Sep 16;42(20):e158. doi: 10.1093/nar/gku849 (PMC4227763; doi:10.1093/nar/gku849)
Supplement: SUPPLEMENTARY DATA [file supp_42_20_e158__index.html]

cnvCapSeq: detecting copy number variation in long-range targeted resequencing data — cnvCapSeq: detecting copy number variation in long-range targeted resequencing data — cnvCapSeq: detecting copy number variation in long-range targeted resequencing data — SUPPLEMENTARY DATA 

# cnvCapSeq: detecting copy number variation in long-range targeted resequencing data

## SUPPLEMENTARY DATA

**Files in this Data Supplement:**

- SUPPLEMENTARY DATA
