# Supplementary material for: cnvCapSeq: detecting copy number variation in long-range targeted resequencing data
Source: Nucleic Acids Res. 2014 Sep 16;42(20):e158. doi: 10.1093/nar/gku849 (PMC4227763; doi:10.1093/nar/gku849)
Supplement: SUPPLEMENTARY DATA [file supp_gku849_nar-02095-met-k-2014-File007.pdf]

Supplementary Material

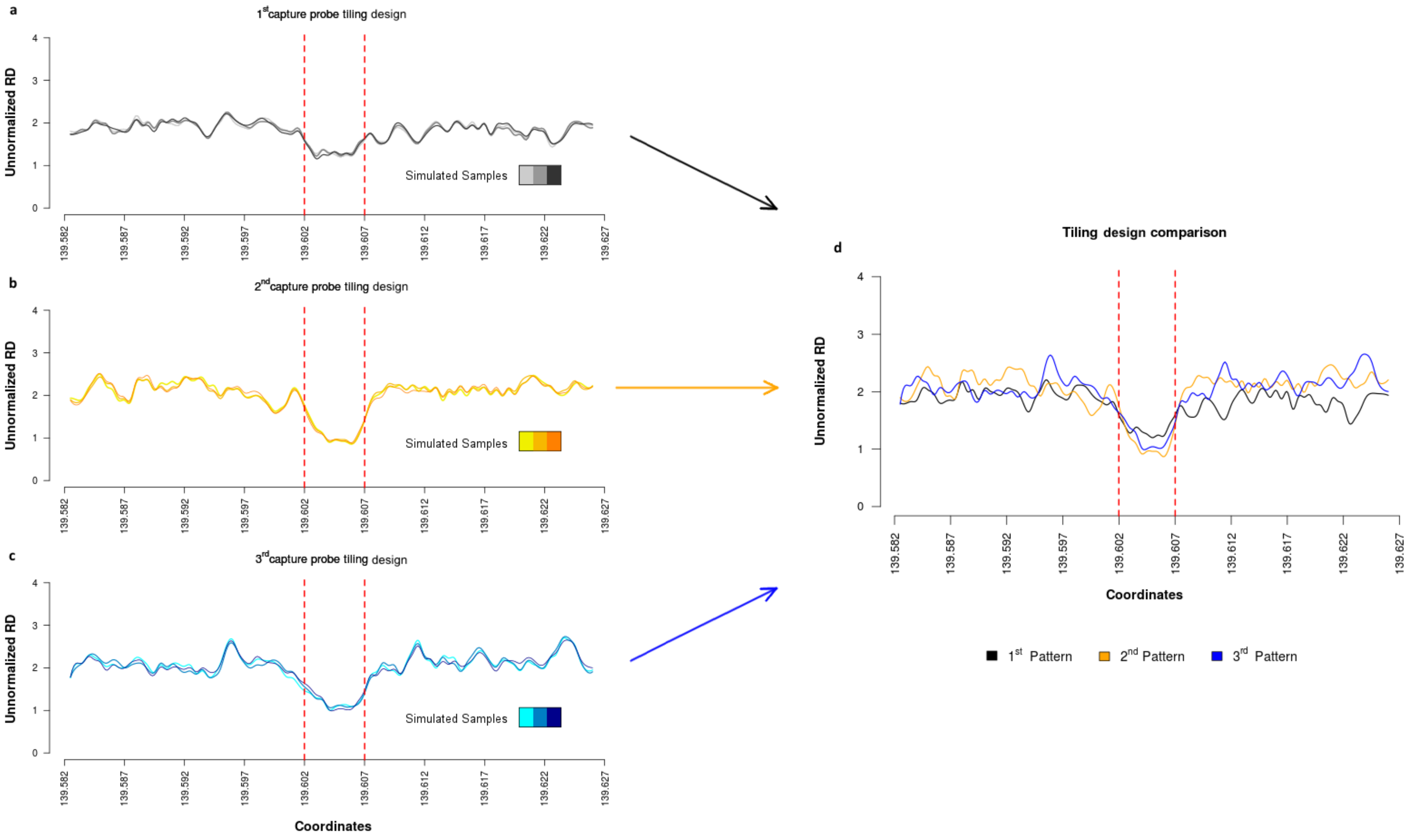

Supplementary Figure 1: Effects of bait design and tiling on read depth of simulated locus

We designed 3 alternative ways to cover a target locus (chr6:139,582,000-139,627,000) with hybridization probes. By varying the probe length and the amount of overlap among probes we demonstrate how bait design can influence the resulting read depth pattern. The red dashed lines denote a known region of low alignability. (a-c) Each experiment (panel) has a unique probe tiling design, which was used to simulate sequence data for 3 different samples, denoted by different color shades. The read depth patterns show a very high degree of similarity within experiments. (d) Representative samples from each tiling design are compared to each other. The noise patterns don't appear to be consistent across experiments.

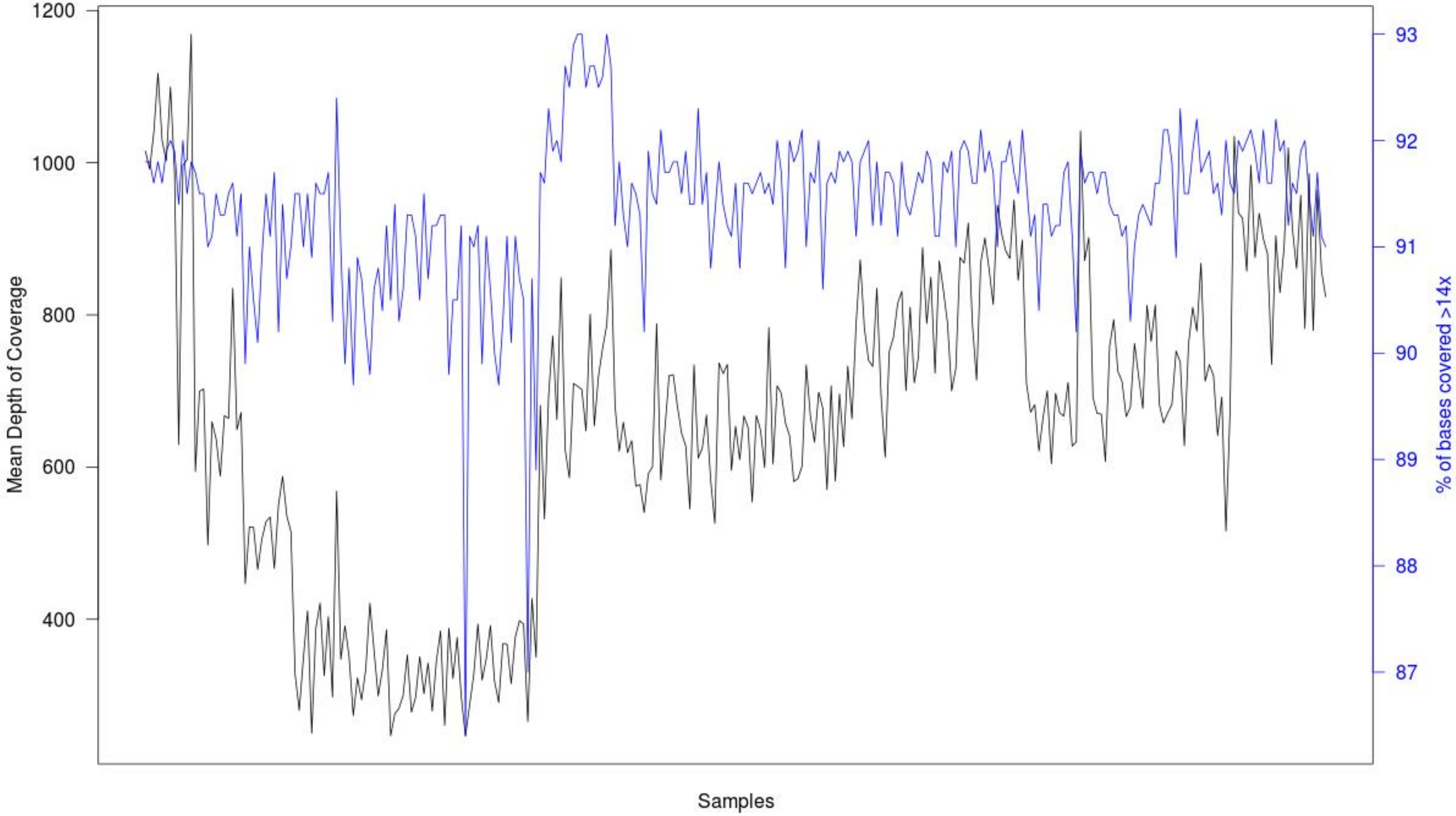

Supplementary Figure 2: Depth of Coverage (DOC) statistics across all 285 samples.

The mean DOC for each sample is represented in black, while the percentage of bases covered at least 14x are represented in blue.

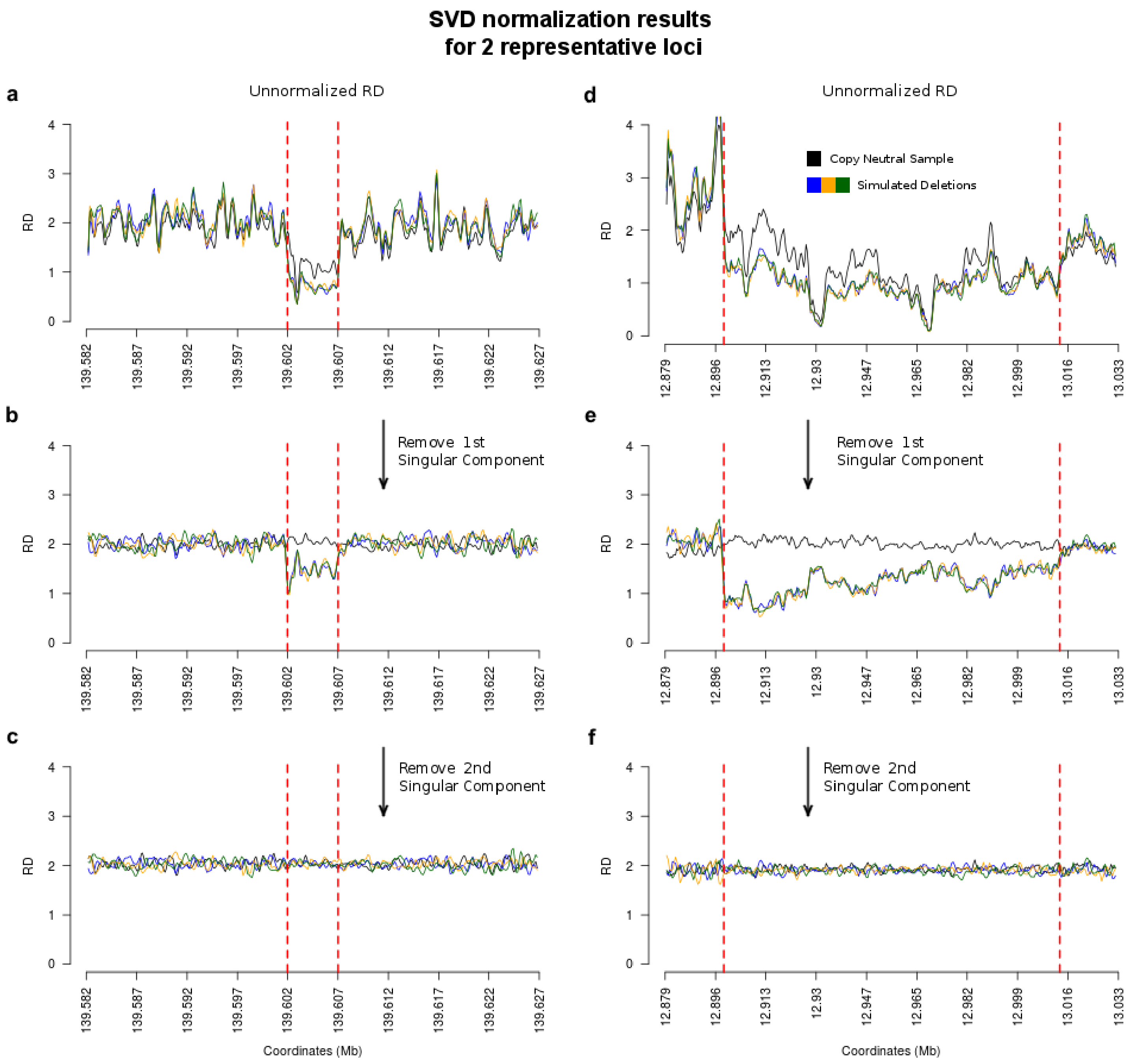

Supplementary Figure 3: SVD normalization results for 2 representative simulation loci

Each column represents a single locus where a deletion was simulated. 4 samples are depicted in each plot: 1 copy-neutral and 3 harbouring heterozygous deletions. The red dashed lines denote the breakpoints of the simulated deletion. Removing the first singular component separates the copy-neutral from the deletion-bearing samples. When further components are eliminated all useful signal appears to be lost. (a-c) 5kb deletion simulated on chromosome 6 (chr6:139,582,000-139,627,000). (d-f) 115kb deletion simulated on chromosome 1 (chr1:12,879,000-13,033,000)

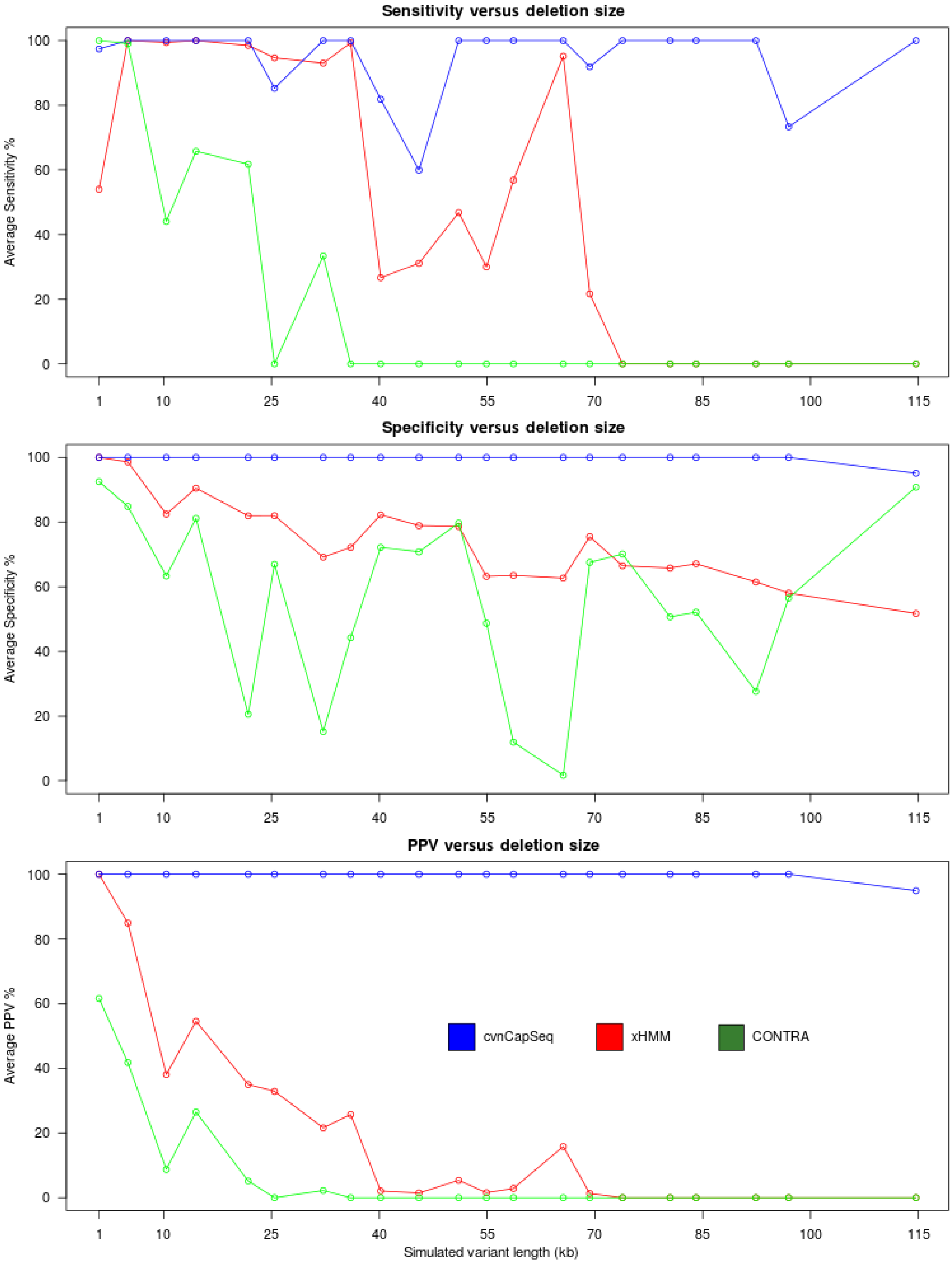

Supplementary Figure 4: Method performance versus simulated deletion size

Sensitivity, specificity and positive predictive value (PPV) across 21 deletion lengths. The performance metrics were averaged across simulated population frequencies. For xHMM and CONTRA performance deteriorates with increasing size, while cnvCapSeq remains consistent throughout.

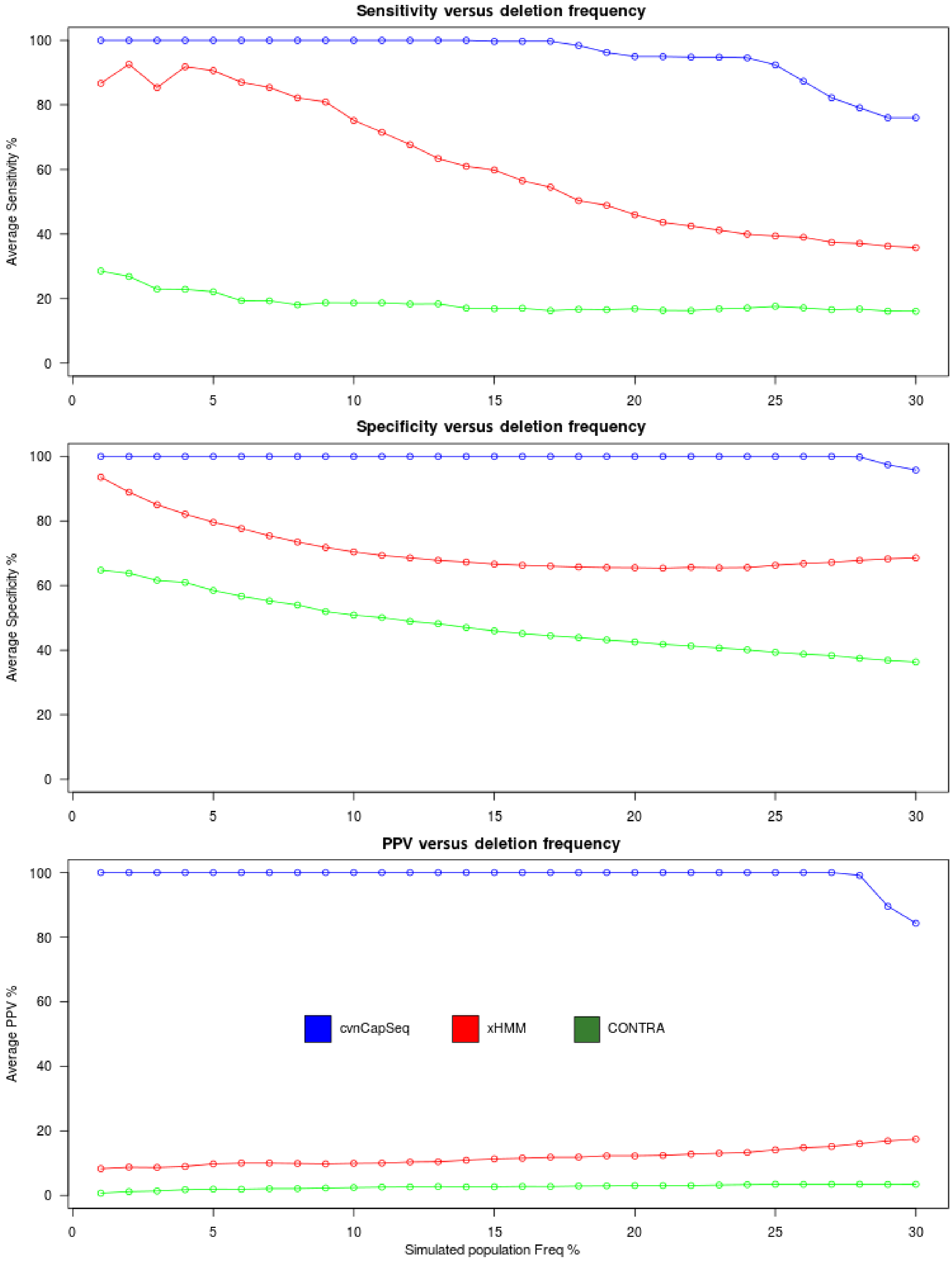

Supplementary Figure 5: Method performance versus simulated population frequencies

Sensitivity, specificity and positive predictive value (PPV) across population frequencies. The cohort size was kept constant at 100 samples and frequencies up to 30% were simulated. The performance metrics were averaged across simulated deletion sizes. Performance declines with increasing frequency for all methods, but xHMM and CONTRA deteriorate earlier and faster than cnvCapSeq.

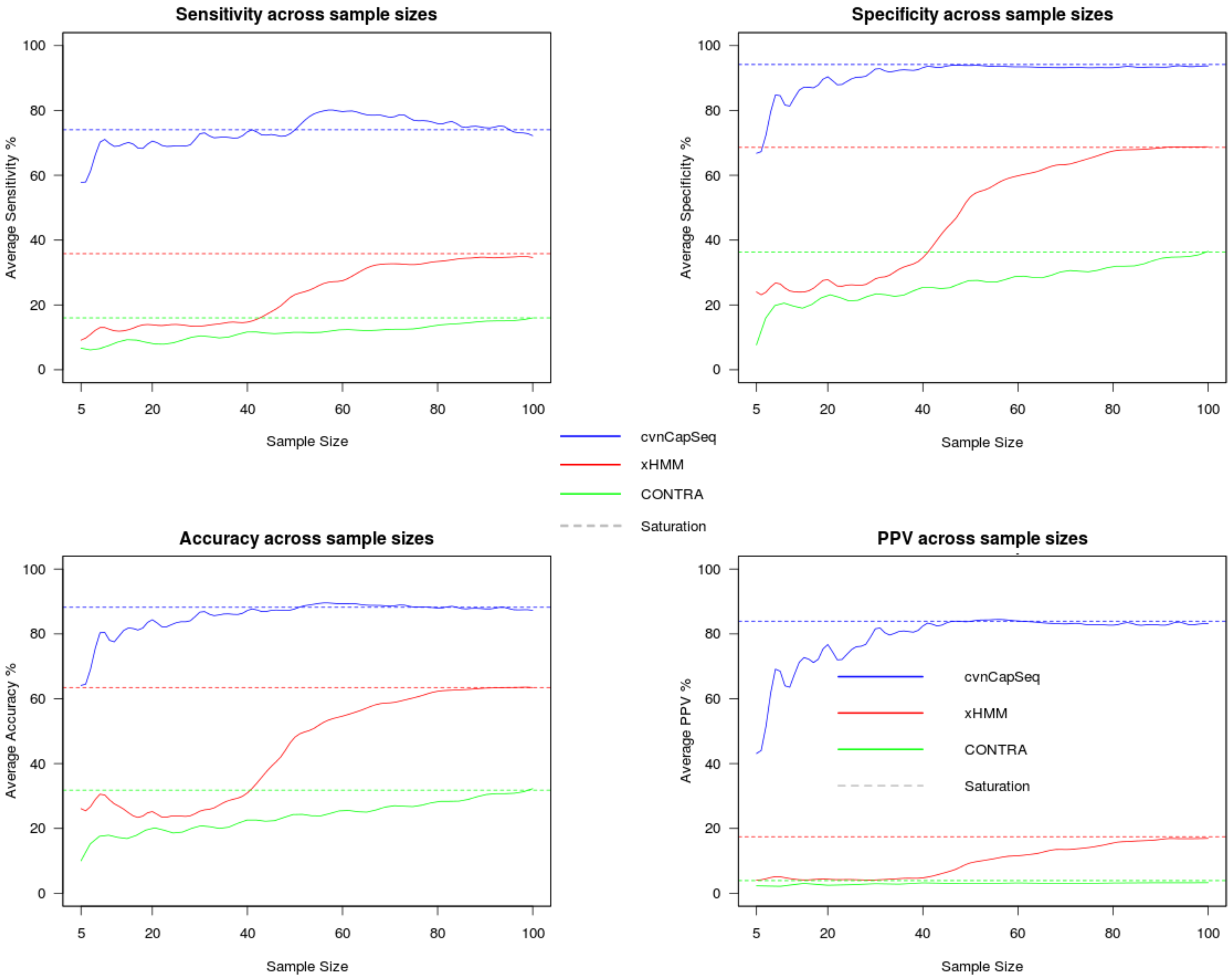

Supplementary Figure 6: Method performance across simulated cohort sizes

Sensitivity, specificity, accuracy and positive predictive value (PPV) across cohort sizes. The CNV population frequency was kept constant at approximately 30%, while cohort sizes from 5 to 100 samples were simulated. The dashed lines denote the performance for the full-sized cohort. cnvCapSeq outperforms xHMM across cohort sizes, with the difference being more pronounced for smaller cohorts. Also, cnvCapSeq achieves saturation faster than xHMM and thus requires smaller cohorts for optimal performance.

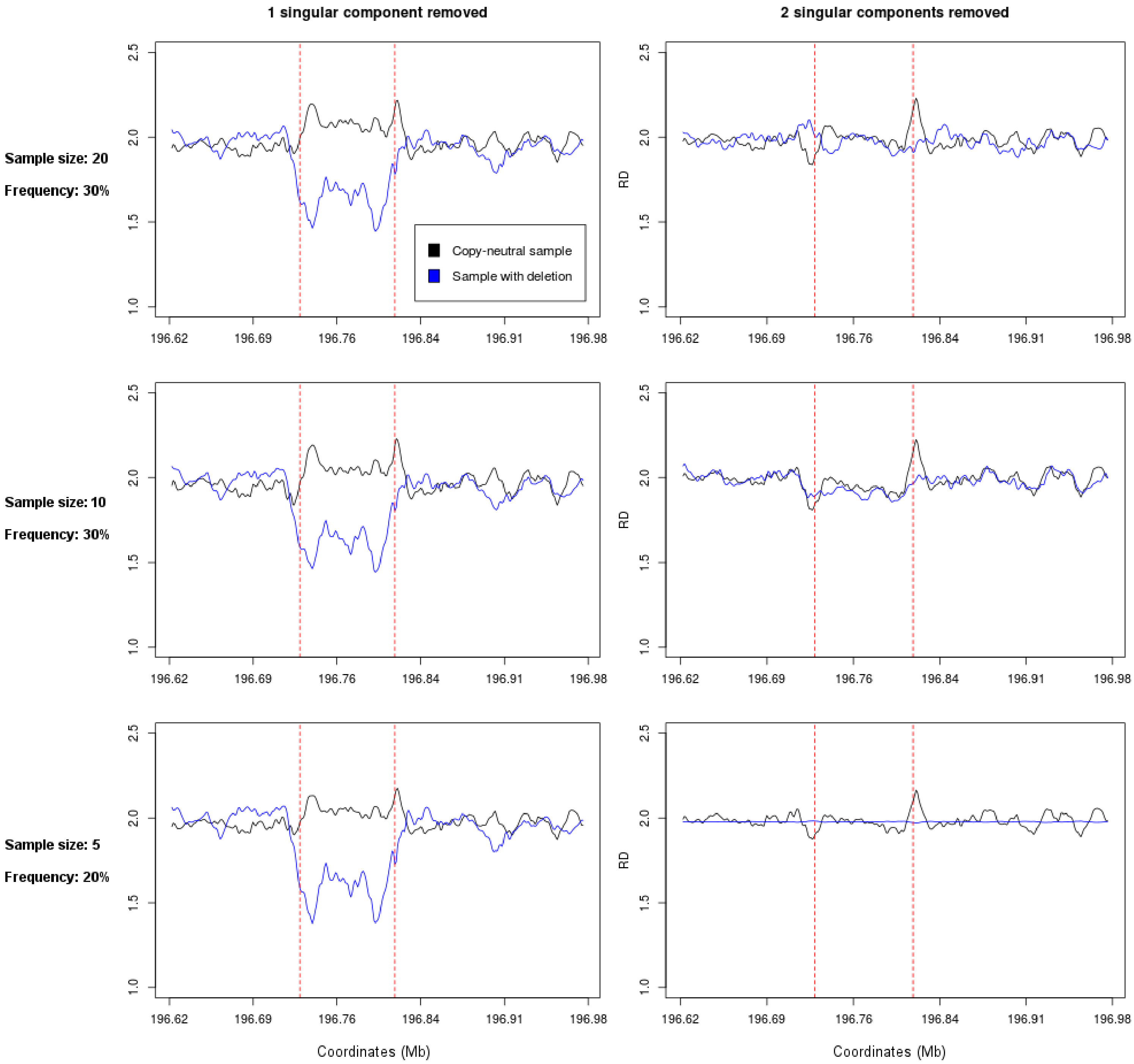

Supplementary Figure 7: Comparison of SVD normalization results for small datasets derived from the RCA cohort.

Each row represents a subset of our original dataset containing copy-neutral samples and samples with validated deletions. The number of samples with deletions is varied in each subset to achieve the desired population frequency. Each column represents the results of the normalization with different numbers of singular components removed. Only one representative copy-neutral sample (CHH1008) and one validated deletion (CHH1039) are plotted in each case.

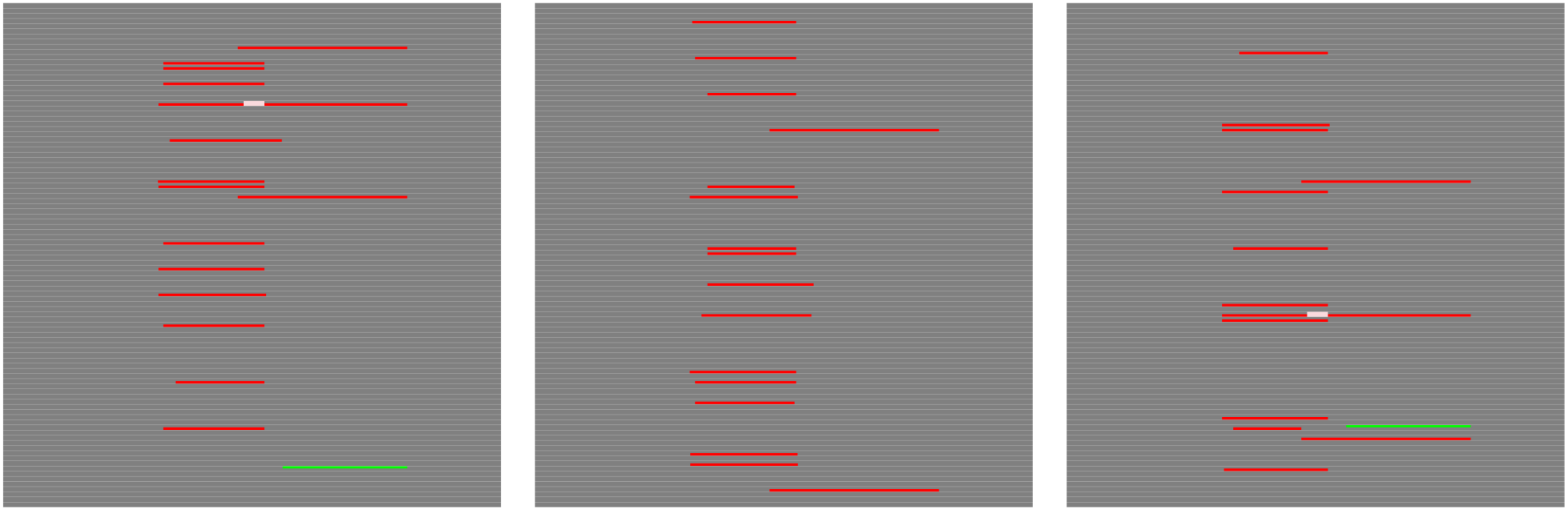

Supplementary Figure 8: Graphical representation of cnvCapSeq's results for all 285 RCA samples across the capture region.

Each (grey) line represents a single sample. Grey color denotes copy-neutral, red color denotes heterozygous deletion, pink denotes homozygous deletion and green corresponds to 3 copies.

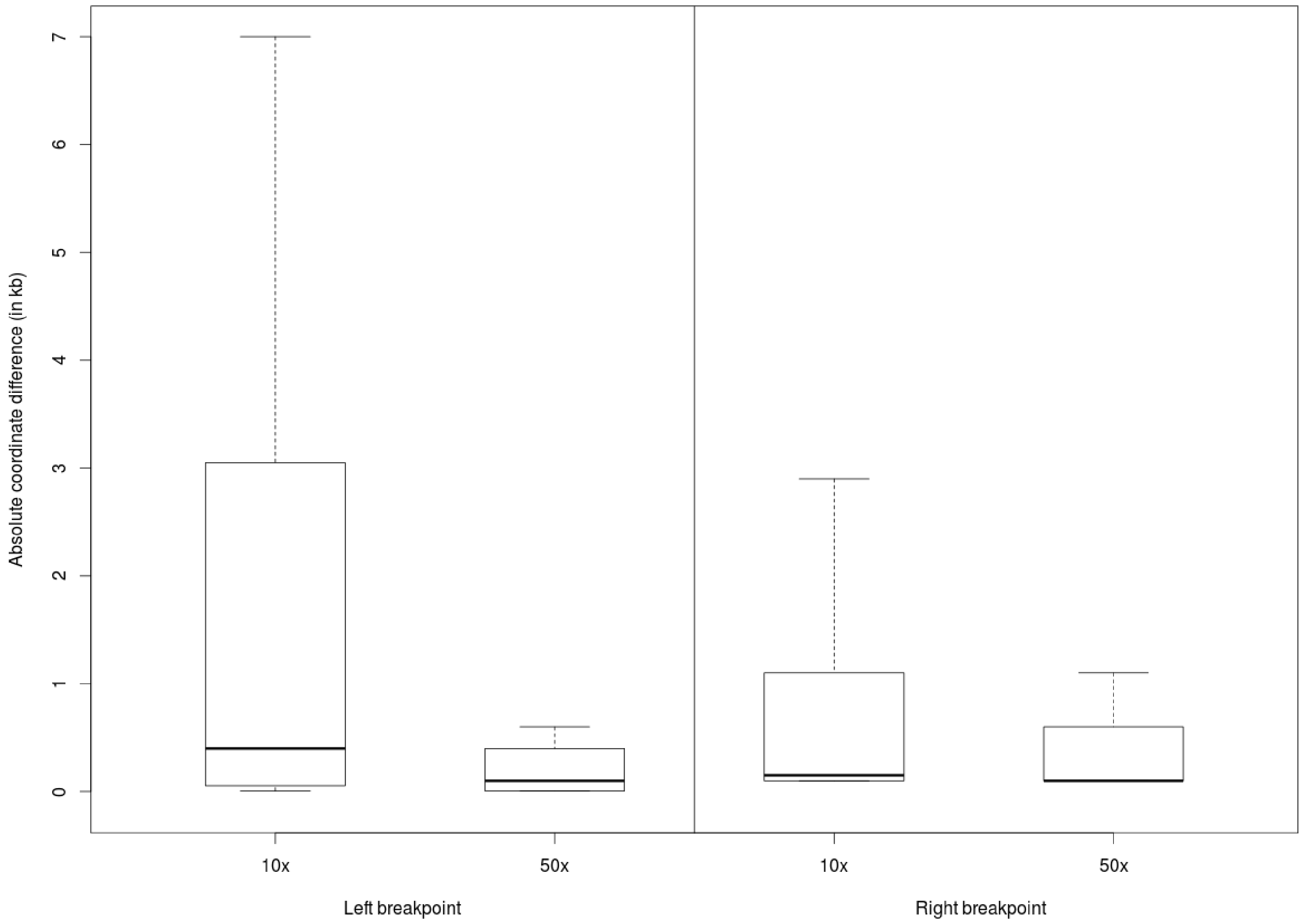

Supplementary Figure 9: Comparison of detected CNV breakpoints between the original and downsampled datasets.

The boxplots summarize the absolute coordinate difference of the detected CNV breakpoints in each downsampled dataset (50x, 10x) versus the original CNV calls.

Supplementary Table 1: 21 genomic loci used in our simulations

Chr	Outer Start Coordinate	Outer End Coordinate	Left CNV breakpoint	Right CNV breakpoint	CNV Length (bp)
1	12,879,000	13,033,000	12,898,653	13,013,316	114663
1	49,895,000	50,019,000	49,914,513	49,998,578	84065
1	72,746,000	72,832,000	72,766,323	72,811,839	45516
1	106,144,000	106,235,000	106,164,396	106,215,440	51044
1	112,672,000	112,726,000	112,691,801	112,706,300	14499
1	152,536,000	152,608,000	152,555,542	152,587,742	32200
1	158,435,000	158,534,000	158,455,170	158,513,839	58669
1	189,298,000	189,403,000	189,317,522	189,383,122	65600
1	196,713,000	196,834,000	196,733,401	196,813,850	80449
1	246,354,000	246,467,000	246,373,568	246,447,415	73847
6	29,841,000	29,936,000	29,860,857	29,915,764	54907
6	31,200,000	31,333,000	31,220,482	31,312,868	92386
6	31,336,000	31,473,000	31,356,165	31,453,117	96952
6	32,470,000	32,546,000	32,490,263	32,526,276	36013
6	66,989,000	67,069,000	67,008,728	67,048,916	40188
6	74,572,000	74,622,000	74,592,060	74,602,439	10379
6	77,417,000	77,479,000	77,437,226	77,458,987	21761
6	78,947,000	79,056,000	78,967,194	79,036,475	69281
6	95,173,000	95,214,000	95,193,322	95,194,336	1014
6	103,717,000	103,783,000	103,737,464	103,762,889	25425
6	139,582,000	139,627,000	139,601,876	139,606,900	5024

Supplementary Table 2: qPCR primers used for the RCA validation.

Gene	Chromosome	Coordinate (start)	Strand	Primer sequence (5'-3')
CFHR3	1	196,749,063	Forward	GGAGAAAGGCTGGTCTCCTACT
		196,749,149	Reverse	CTGAGACTGTCGTCCGTGTTAC
CFHR1	1	196,796,120	Forward	AAATGCAGGTCCACTGGTAAGT
		196,796,340	Reverse	GAGATGATGATGCTACCGGTTT
LOC100996886	1	196,842,723	Forward	TGACTGGTGACTCATTCCTCTG
		196,842,826	Reverse	TCAGATAGGGTTGGCCTTTCTA
CFHR4	1	196,865,825	Forward	ACGATCCAAGTCATCCCTAGAA
		196,865,910	Reverse	TGGAATCTGACTCCTCACCTTT

Supplementary Table 3: Overall performance comparison across methods for the simulated dataset

	Sensitivity	Specificity	PPV	NPV	Accuracy
**cnvCapSeq**	92.08%	99.77%	98.37%	98.80%	98.75%
**xHMM**	48.27%	70.47%	12.29%	94.08%	68.71%
**CONTRA**	17.24%	46.24%	2.84%	85.98%	43.82%
**CoNIFER**	0.02%	95.29%	0.07%	86.08%	82.57%

Supplementary Table 4: cnvCapSeq-generated CNV calls for the RCA cohort along with the genes affected.

Sample	Chr	Start Coordinate	End Coordinate	Length	Copy Number	Overlaps		
						CFHR3	CFHR1	CFHR4
CHH1039	1	196,734,200	196,810,800	76,600	1	●	●	
CHH1045	1	196,811,300	196,905,000	93,700	3			●
CHH1130	1	196,734,200	196,804,900	70,700	1	●	●	
CHH1141	1	196,734,200	196,810,800	76,600	1	●	●	
CHH1159	1	196,727,100	196,810,900	83,800	1	●	●	
CHH1164	1	196,723,700	196,804,900	81,200	1	●	●	
CHH1181	1	196,723,700	196,804,900	81,200	1	●	●	
CHH1190	1	196,786,900	196,905,000	118,100	1		●	●
CHH1192	1	196,723,700	196,804,900	81,200	1	●	●	
CHH1193	1	196,734,200	196,810,900	76,700	1	●	●	
CHH1201	1	196,734,200	196,819,600	85,400	1	●	●	
CHH1232	1	196,723,700	196,790,800	181,300	1	●		
	1	196,790,900	196,804,900		0		●	
	1	196,805,000	196,905,000		1			●
CHH1236	1	196,723,700	196,804,900	81,200	1	●		
CHH1239	1	196,723,700	196,804,900	81,200	1	●		
CHH1240	1	196,726,800	196,804,900	78,100	1	●		
CHH1249	1	196,786,900	196,905,000	118,100	1		●	●
CHH1278	1	196,786,900	196,905,000	118,100	1		●	●

CHH1285	1	196,727,100	196,811,000	83,900	1	●	●	
CHH1420	1	196,723,700	196,804,900	81,200	1	●	●	
CHH1424	1	196,723,700	196,804,900	81,200	1	●	●	
CHH1437	1	196,732,500	196,804,900	72,400	1	●	●	
CHH1443	1	196,734,200	196,819,600	85,400	1	●	●	
CHH1465	1	196,734,200	196,804,900	70,700	1	●	●	
CHH1478	1	196,734,200	196,804,900	70,700	1	●	●	
CHH1489	1	196,786,900	196,905,000	118,100	1		●	●
CHH1512	1	196,734,200	196,804,900	70,700	1	●	●	
CHH1519	1	196,723,700	196,804,900	81,200	1	●	●	
CHH1526	1	196,723,700	196,804,900	81,200	1	●	●	
CHH1530	1	196,734,200	196,810,800	76,600	1	●	●	
CHH1560	1	196,786,900	196,905,000	118,100	1		●	●
CHH1562	1	196,734,200	196,783,100	48,900	1	●		
	1	196,819,800	196,905,000	85,200	3			●
CHH1564	1	196,723,700	196,804,900	81,200	1	●	●	
CHH1583	1	196,723,700	196,804,900	81,200	1	●	●	
CHH1584	1	196,723,700	196,790,800	181,300	1	●		
	1	196,790,900	196,804,900		0		●	
	1	196,805,000	196,905,000		1			●
CHH1586	1	196,734,200	196,810,800	76,600	1	●	●	
CHH1613	1	196,727,000	196,804,900	77,900	1	●	●	
CHH1624	1	196,726,800	196,804,900	78,100	1	●	●	
CHH1626	1	196,786,900	196,905,000	118,100	1		●	●
CHH1645	1	196,723,700	196,804,900	81,200	1	●	●	
CHH1646	1	196,726,900	196,811,100	84,200	1	●	●	
CHH2009	1	196,734,200	196,804,900	70,700	1	●	●	
